# Phytogenic silver nanoparticles from tissue-cultured *Kaempferia angustifolia* — an underutilized medicinal herb: a comparative antibacterial study on urinary pathogens

**DOI:** 10.1186/s43141-022-00414-4

**Published:** 2022-09-08

**Authors:** Avijit Chakraborty, Sk Moquammel Haque, Diganta Dey, Swapna Mukherjee, Biswajit Ghosh

**Affiliations:** 1 Plant Biotechnology Laboratory, Department of Botany, Ramakrishna Mission Vivekananda Centenary College, Rahara, Kolkata, 700118 India; 2 Department of Botany, East Calcutta Girls’ College, Lake Town, Kolkata, 700089 India; 3 Department of Microbiology, Ashok Laboratory Clinical Testing Centre Private Limited, Kolkata, 700068 India; 4 Department of Microbiology, Dinabandhu Andrews College, Garia, Kolkata, 700084 India

**Keywords:** *Kaempferia angustifolia*, Antimicrobial activity, Nanoparticle, Multidrug resistant, Urinary tract infection

## Abstract

**Background:**

Ethnomedicinally important *Kaempferia angustifolia* is a rhizomatous aromatic herb belonging to the family Zingiberaceae. The present manuscript deals with the green synthesis of silver nanoparticles through a rapid reduction process mediated by the rhizome extract of tissue culture-raised plants. The present study was conducted to evaluate the antimicrobial activity of the bio-nanoparticles, and the plant extracts themselves against seven multidrug-resistant urinary tract infecting (MDR-UTI) pathogens.

**Result:**

The ethanolic extracts of the rhizomes of the plant executed a very rapid synthesis of silver bio-nanoparticles, and the generation of the nanoparticles was confirmed through UV-vis spectrophotometry, dynamic light scattering (DLS), and electron dispersion spectroscopic (EDS) analysis. Finally, the precise shapes and dimensions of these nanoparticles were confirmed under the transmission electron microscope (TEM). The shapes of the nanoparticles obtained were diverse in nature and varied from rod, triangular, spherical, to oval shaped, with the size, ranging from 10–60 nm. Silver nanoparticles exhibited a maximum zone of inhibition (ZI) of 16.93 ± 0.04 mm against isolate no. 42332. The ex vitro and in vivo extracts exhibited *ZI* 14.03 ± 0.04 mm and 11.56 ± 0.04 mm, respectively, against the same strain, which are comparatively lower than the nanoparticles but unignorable.

**Conclusion:**

Although the pathogens used in the present study are resistant to at least three or more types of pharmacologically important antibiotics, nanoparticles, as well as the plant extracts, exhibited significant inhibition to all the seven MDR-UTI pathogens, which confirms that they are highly antimicrobic. Hence, this underutilized medicinal plant extracts of *K. angustifolia* and the bio-nanoparticles synthesized from these can be explored in pharmaceutical industries to treat multidrug-resistant human pathogenic bacteria. Furthermore, their broad-spectrum activity leads to the opportunity for the synthesis of future generation drugs.

**Supplementary Information:**

The online version contains supplementary material available at 10.1186/s43141-022-00414-4.

## Background

Medicinal plants are always a potential source of bioactive compounds and have been used in the very past to treat pathogenic bacterial strains [1]. *Kaempferia angustifolia* Roscoe is a member of the Zingiberaceae family with versatile ethnomedicinal importance. The genus *Kaempferia* is native to Southeast Asia and is grown wildly throughout India, Indonesia, and Thailand. More than 50 species are reported under this genus, but only a few of them, like *K. galanga*, *K*. *rotunda*, and *K. parviflora*, are explored until date. *K. angustifolia*, with flavonoids as one of the major active compounds, has not yet been appropriately explored [2, 3]. Additionally, the rhizomes of *K. angustifolia* are known for their high nutritional value and good antioxidant properties [4]. The rhizomes of this species have traditionally been used as a medication for the common cold, fever, etc. [5]. This plant also contains high-value essential oil, which contains a diverse group of phyto-compounds from which only a few are identified [6, 7].

Nanotechnology combines nanoscience and biotechnology and has gained popularity in the past few years. Green synthesis of the bio-nanoparticles, alternatively called phytogenic nanoparticles, is widely becoming a primary research interest because of its non-toxicity [8]. The particle with a size ranging from 1 to 100 nm has a wide range of applications in health care, cosmetics, biomedical sciences, chemical industries, electronics, single-electron transistors, light emitters, and nonlinear optical devices [9]. Nanotechnology is also growing in interest for its targeted drug delivery with the correct dose against severe diseases like tumours, cancer, and HIV [10]. Silver bio-nanoparticles are mostly used as a drug against microbes due to specific killing mechanisms, like creating holes in the membrane with less grown resistance [11]. Bio-nanoparticles could be an alternative to commercially used antibiotics for those multidrug-resistant strains and are also regarded as next-generation antibiotics [12]. The *K. pandurata*, *K. rotunda*, *K. galanga*, and *K. parviflora* were previously studied for nanoparticle production [13–16]. However, according to our knowledge, no such attempt has been made with *K. angustifolia* in nanobiotechnology.

Nowadays, urinary tract infection is a major concern worldwide, including in India. About 10% of people worldwide and 24% in India are infected with both symptomatic and asymptomatic UTI bacteriuria [17]. UTI in pregnant women also causes a high morbid condition for both mother and fetus [18]. Extensive use, as well as misuse of antibiotics, makes bacteria resistant to them. Therefore, multidrug-resistant bacteria is a primary concern in the present day [19, 20]. Bacteria can develop resistance by acquiring different development strategies like efflux pumps and mutation in their genome, etc. [21, 22]. The antimicrobial activity of other species of the genus *Kaempferia* was extensively explored by some researchers [16, 23–25]. However, only two reports are available on the antimicrobial activity of *K. angustifolia* against laboratory-grade nonpathogenic standard strain [7, 26]. Nevertheless, according to our knowledge, no such attempt has yet been made against pathogenic drug-resistant strain.

The present study focused on the following: (1) the rapid production of silver bio-nanoparticles through catalysis of silver salt by using *K. angustifolia* rhizome extract, (2) the investigation of the ethanolic extract of the rhizome against MDR human UTI pathogenic bacteria to screen out the potency of the extract over conventional antibiotics, and (3) the screening of antimicrobial potency of the synthesized bio-nanoparticles against those bacterial strains where commercially approved drugs are insensitive.

## Methods

### Plant material

**Fig. 1:**
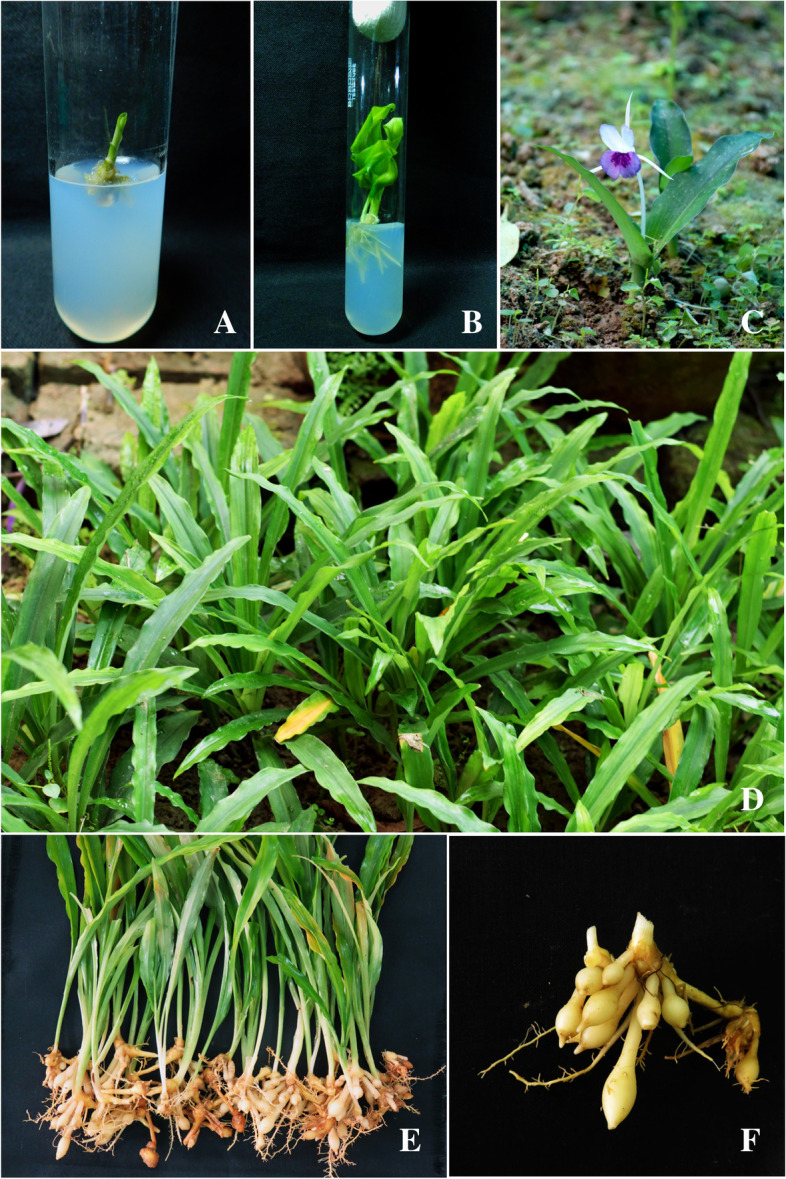
The plant *Kaempferia angustifolia*. **A** In vitro inoculation of the plant. **B** In vitro propagation of the plant. **C** Ex vitro flowering of the plant after acclimatization. **D** Ex vitro mature plant. **E** Harvested ex vitro plant. **F** Ex vitro grown rhizome

In vitro propagated plants were successfully acclimatized and transferred to the field for proper growth according to our previous article (Fig. 1 A and B) [2]. Identification of the source plant has been made from the Central National Herbarium (CNH) of the Botanical Survey of India (BSI), Howrah, India (voucher no. ‘CNH/87/2012/Tech.II/912’ and plant specimen no. ‘RKMVC-Ka-1’). After 5 months of field transfer, flowering was observed (Fig. 1C), and after 4 months of flowering (Fig. 1D), the rhizomes were harvested from an ex vitro (tissue-cultured plant after field transfer) mature plant at the age of 9 months. In addition, rhizomes of naturally grown in vivo (Fig. 1E) plants were also collected at the same age. Both in vivo and ex vitro rhizomes were further used for extraction and to conduct experiments.

### Extraction of the plant material

The harvested rhizomes (100 g fresh weight) were sun-dried (68.8 g dry weight) and then grinded to powder. Ten grams of this powder was extracted in the Soxhlet apparatus in the presence of ethanol, and finally, 1.80 g dry extract was obtained. The extracted material was used to carry out the synthesis of bio-nanoparticles and to screen out the antimicrobial potency.

### Isolation and identification of UTI bacterial pathogens

Bacterial pure colonies from the urine sample of the diseased patient were collected by plating the samples on MacConkey agar and nutrient agar plates (Himedia®, India) at a temperature of 37 ± 2 °C for 24 h (Ashok Laboratory Clinical Testing Centre Private Limited, Kolkata 700068, India, a BSL-3 laboratory and NABL accredited). The VITEK 2 Compact system bioMerieux machine was used to identify the pathogenic strains. Initial identification was done by conducting the following investigations — oxidase activity, catalase activity, indole test, methyl red, citrate test, Voges-Proskauer test, and hydrogen sulphide production.

### Synthesis of bio-nanoparticle

The rhizome extract of both in vivo and ex vitro plants of *K. angustifolia*, in four different amounts (1.0, 2.0, 3.0, and 4.0 g), was added separately to 10 ml of 1.0 mM silver nitrate solution; all the experimental solutions were incubated at 45 °C temperature for 10 h under shaken condition (80 rpm). The nanoparticles synthesized were then incubated further at 28 °C for additional 10 h to obtain proper size distribution. As a result, the solutions started to turn sequentially transparent to light yellow to deep yellow to light brown and finally dark brown colour, indicating the formation of silver bio-nanoparticles. Nanoparticles were then stored in dried conditions under low temperatures.

### Characterization of bio-nanoparticles

#### UV-vis spectra analysis

Samples were diluted in Milli-Q water to prepare variant concentrations for better peak detection. Biosynthesized nanoparticles were examined in a UV-vis spectrophotometer (Shimadzu, UV — 1800, UV Spectrophotometer, Japan). The scan was done in a range of 300 to 700-nm wavelength for nanoparticles and 200 to 800 nm for the plant extract to obtain the end peaks.

#### Dynamic light scattering analysis (DLS)

As the plant extract contains several metabolites, synthesized bio-nanoparticles exist in different sizes with coated proteins. Therefore, DLS was done to analyse the average size of the nanoparticles using a Zetasizer Nano ZS90 instrument.

#### High resonance transmission electron microscopy (HRTEM) and energy-dispersive X-ray spectroscopy (EDS) analysis

Carbon-coated copper TEM grids were coated with 1.0 μl of the silver nanoparticle solutions and appropriately dried. A high-resolution transmission electron microscopy (HRTEM: JEM-2100, JEOL, Japan) was used to study the morphology of nanoparticles. In addition, energy-dispersive spectroscopy analysis (EDS) (Oxford INCA instruments) was done to analyse the elemental composition of the nano-silver particles.

### Antimicrobial potency analysis

#### Agar cup method

Agar well diffusion method was used to determine the antibacterial potency of extracts along with the silver bio-nanoparticle synthesized from *K. angustifolia* following the methodology described by Deans and Ritchie (1987) with minor modification [27]. The plant extract was dissolved in dimethyl sulfoxide (DMSO, which is used over here as a negative control) at a concentration of 100, 50, and 25 mg/ml and nanoparticles in water at a concentration of 10, 25, and 50 μg/ml for application on microbial strains. Mueller-Hinton agar (MHA) plates were used to determine the zone of inhibition. The plates were punched with a cork borer of 6.0-mm diameter, and samples (crude plant extract and silver bio-nanoparticle) in different concentrations were poured into the agar cups. After incubation of plates at 37 °C for 24 h, zones of inhibition were measured.

### Minimum inhibitory concentration (MIC) and minimum bactericidal concentration (MBC)

To determine the MIC value, broth dilution method was carried out following Ericsson and Sherris (1971) method with minor modifications [28]. Bacterial inoculums of 100 μl were added to every tube containing plant extract (0.25, 0.50, 0.75, 1.00, 1.25, 1.50, 1.75, 2.00, 2.25, 2.50, 2.75, 3.00, 3.25, 3.50, 3.75, 4.00, 4.25, 4.50, 4.75, 5.00 mg/ml) and nanoparticles at different dilutions (0.25, 0.50, 0.75, 1.00, 1.25, 1.50, 1.75, 2.00, 2.25, 2.50, 2.75, 3.00, 3.25, 3.50, 3.75, 4.00, 4.25, 4.50, 4.75, 5.00, 5.25, 5.50, 5.75, 6.00. 6.25, 6.50, 6.75, 7.00, 7.25, 7.50, 7.75, 8.00, 8.25, 8.50, 9.00, 9.25, 9.50, 9.75, 10.00 μg/ml) which were then incubated at 37 °C for 24 h. The MIC values corresponded to the lowest concentration of the extract and nanoparticle that could prevent bacterial growth. The MBC values were also determined by plating the lowest respective dilutions of the sample used to analyse the MIC. A 100 μl sample of each bacterium was plated for each concentration of the plant crude extract, and bio-nanoparticles and data were collected after 24 h of incubation.

## Result

### Characterization of silver nanoparticles

The nanoparticles synthesized from the plant extract were characterized with UV-vis spectra, dynamic light scattering, transmission electron microscopy, and energy-dispersive X-ray spectroscopic analysis. The shapes, sizes, and the structural configurations of the nanoparticles were also resolved in a decisive manner through the experiments. The stability of the nanoparticles was ascertained by employing the same set of experiments used for characterization, after 5 days of synthesis. No distortion or aggregation was seen even after 5 days of synthesis ensuring their sustainability.

### UV-vis spectra analysis

**Fig. 2:**
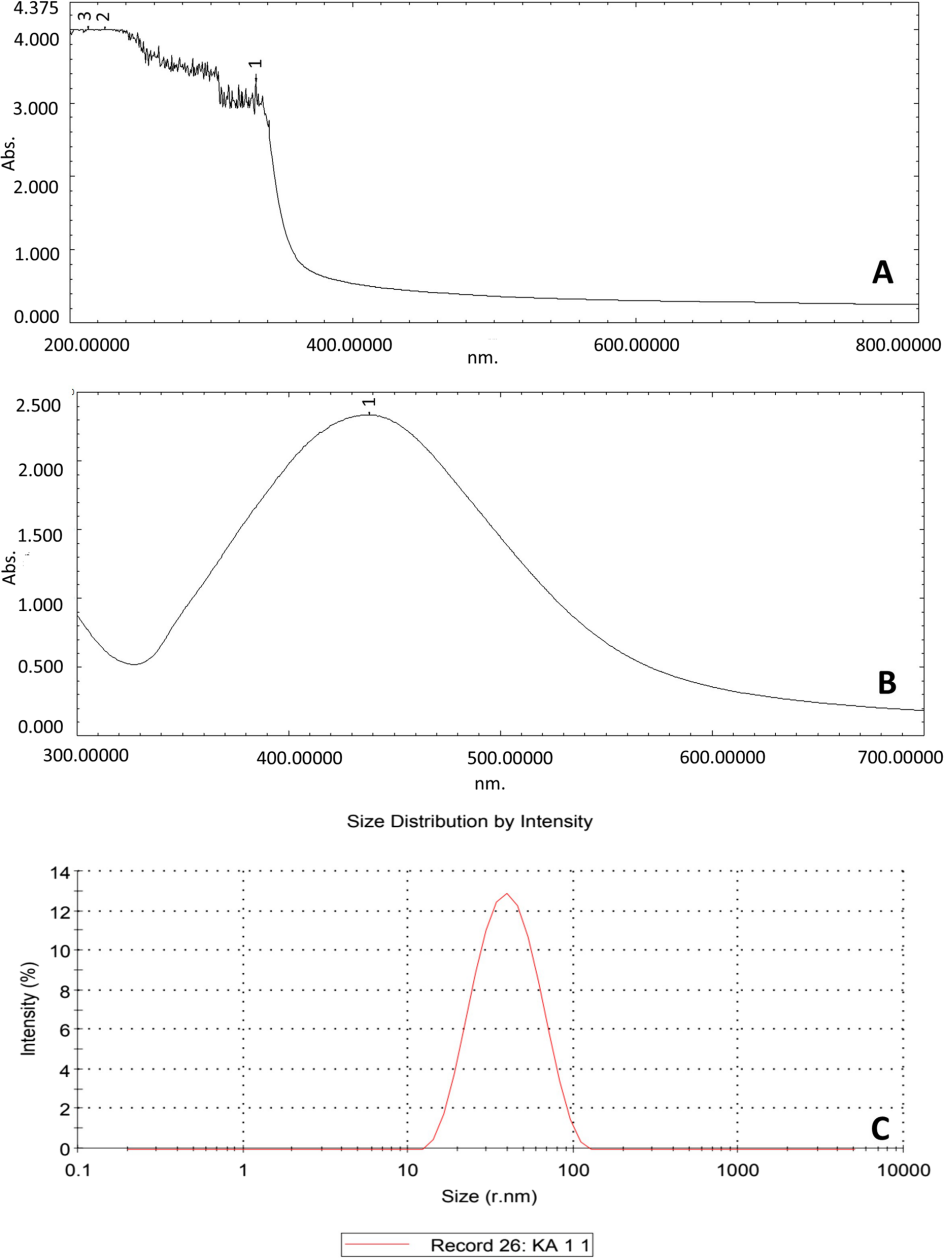
**A** UV-visible spectrum of silver nanoparticle synthesized from *Kaempferia angustifolia* rhizome. **B** UV-visible spectrum of *Kaempferia angustifolia* rhizome extract. **C** DLS spectrum of silver nanoparticle

The colour change from light yellow to the pale brown colour indicated the formation of nanoparticles after 20 h of incubation (first 10 h at 48 °C and last 10 h at 28 °C). The colour changes of the solution occur due to the excitation of the surface plasmon resonance of the silver bio-nanoparticles. The maximum absorbance of the nanoparticles was recorded at 420 nm (Fig. 2A).

### Dynamic light scattering analysis (DLS)

The total thickness of the silver bio-nanoparticles, along with the covering agent, was determined through the DLS instrument. The average size distribution of plant-mediated silver bio-nanoparticles was 36.36 nm with 100% intensity (Fig. 2C).

### High resonance transmission electron microscopy (HRTEM) and energy-dispersive X-ray spectroscopy (EDS) analysis

**Fig. 3:**
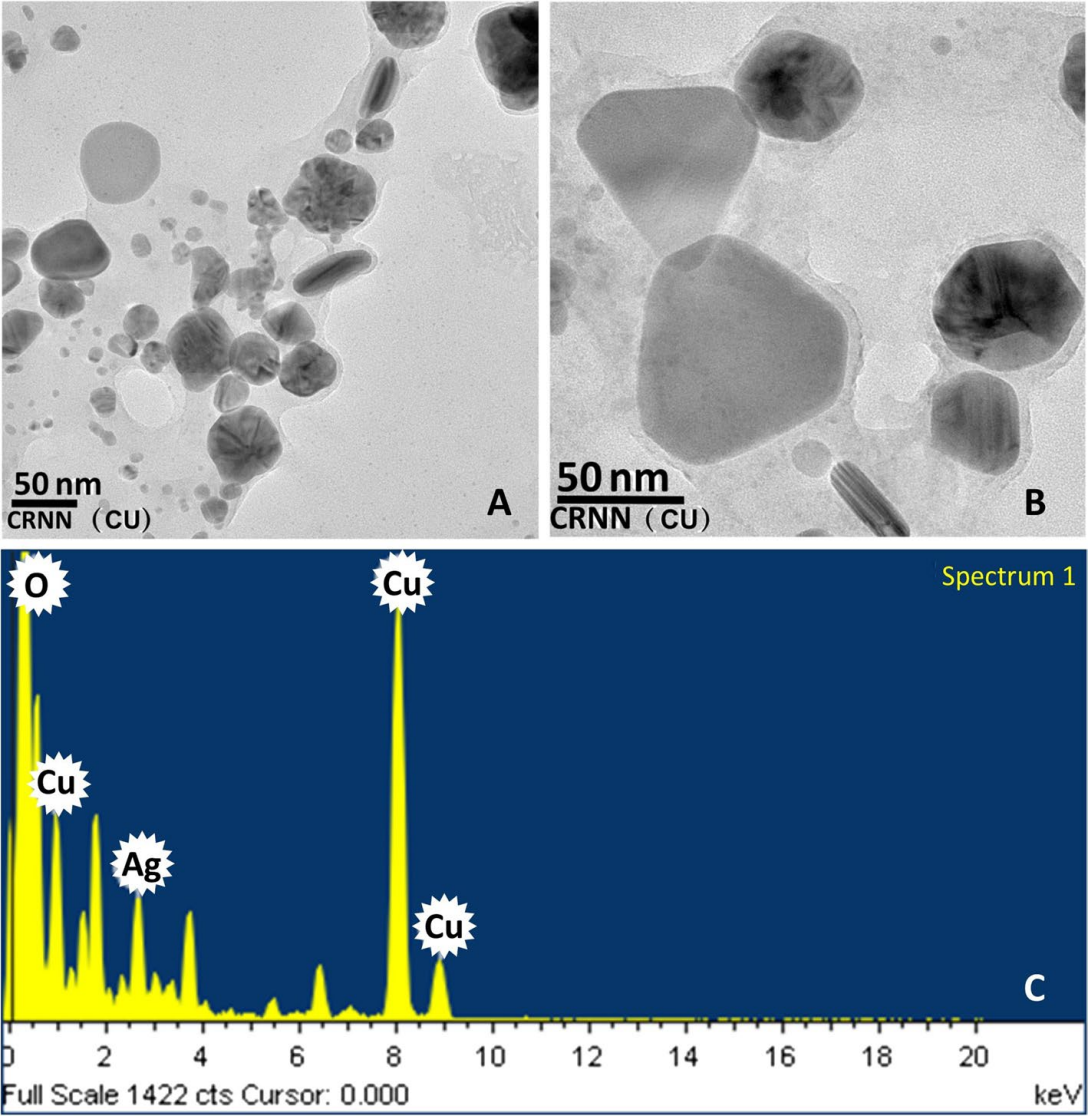
HRTEM image of silver nanoparticle synthesized from *Kaempferia angustifolia*. **A** Size distribution of nanoparticles. **B** Shape distribution of nanoparticles. **C** EDS spectrum of silver nanoparticles shows the presence of silver

The actual size distribution along with the shape of the silver bio-nanoparticles was analysed by HRTEM. The size of the bio-nanoparticles ranged between 10 to 50 nm in diameter (Fig. 3A). Various shapes of the particles like trigonal, hexagonal, rod, oval, and spherical were observed (Fig. 3B). The percentage of weight and atomics of the silver bio-nanoparticles were determined by EDS spectrum 13.63 and 6.07, respectively (Fig. 3C).

### Antibacterial activity

The clinical isolates *Escherichia coli* (isolate numbers — 42571, 42423), *Klebsiella pneumoniae* (isolate numbers — 42269, 43164), *Pseudomonas aeruginosa* (isolate numbers — 42230, 42332), and *Staphylococcus saprophyticus* (isolate number — 43546) were used to study the potency of the bio-nanoparticles as well as the plant extracts. The seven UTI strains were also screened for resistance/susceptibility against 15 commercially important antibiotics (Table 1).

**Table 1:** Antimicrobial resistance of twenty pathogenic bacteria against 15 well-known antibiotics

Pathogen	Patient profile		Biochemical characteristics	Antibiotics					
				Amoxicillin	Amikacin	Aztreonam	Ceftriaxone	Cefuroxime	Ciprofloxacin
	Age (years)	Sex							
Isolate no. 42571	65	F	Ox (−), Cat (+), Ind (+), MR (+), Cit (−), VP (−), H_2_S (−)	R	S	R	R	R	R
Isolate no. 42423	50	M	Ox (−), Cat (+), Ind (+), MR (+), Cit (−), VP (−), H_2_S (−)	S	S	R	R	R	−
Isolate no. 42269	72	M	Ox (−), Cat (+), Ind (−), MR (−), Cit (+), VP (+), H_2_S (−)	R	R	R	R	R	I
Isolate no. 42230	75	M	Ox (+), Cat (+), Ind (−), MR (−), Cit (+), VP (−), H_2_S (−)	−	S	R	−	I	I
Isolate no. 43546	2 y 10 m	F	Ox (−), Cat (+), Ind (−), MR (−), Cit (−), VP (−), H_2_S (+)	S	S	S	S	R	S
Isolate no. 43164	70	F	Ox (−), Cat (+), Ind (−), MR (−), Cit (+), VP (+), H_2_S (−)	R	R	R	R	R	R
Isolate no. 42332	32	F	Ox (+), Cat (+), Ind (−), MR (−), Cit (+), VP (−), H_2_S (−)	I	S	R	R	R	R

Pathogen	Antibiotics								
	Piperacillin	Cefoperazone	Ceftazidime	Ofloxacin	Sulbactam	Nitrofurantoin	Doxycycline	Norfloxacin	Meropenem
Isolate no. 42571	S	R	R	R	S	S	S	R	S
Isolate no. 42423	S	S	R	R	R	R	R	R	S
Isolate no. 42269	R	R	R	I	R	−	S	I	I
Isolate no. 42230	S	−	R	R	R	R	S	S	S
Isolate no. 43546	S	S	S	S	I	R	R	I	S
Isolate no. 43164	R	R	R	R	−	R	R	−	R
Isolate no. 42332	R	R	S	S	S	I	S	R	I

*R* resistant, *S* sensitive, *I* intermediate, ‘−’ not tested, *F* female, *M* male. Isolate no. 42571, *Escherichia coli*; isolate no. 42423, *Escherichia coli*; isolate no. 42269, *Klebsiella pneumoniae*; isolate no. 42230, *Pseudomonas aeruginosa*; isolate no. 43546, *Staphylococcus saprophyticus*; isolate no. 43164, *Klebsiella pneumoniae*; isolate no. 42332, *Pseudomonas aeruginosa*. *Ox* oxidase test, *Cat* catalase test, *Ind* indole test, *MR* methyl red test, *Cit* citrate test, *VP* Voges-Proskauer test, *H*_*2*_*S* hydrogen sulphide

### Identification and screening for resistance and susceptibility

Identification of the bacterial strains was done through biochemical characterization in the VITEK 2 Compact system bioMeriuex machine as well as manually through oxidase, catalase, indole, methyl red, citrate, Voges-Proskauer, and hydrogen sulphide production analysis (Table 1). Antibiotic susceptibility and resistance of the microorganism used in the present study are also studied through VITEK 2 system in the presence of the antibiotics amoxicillin, amikacin, aztreonam, ceftriaxone, cefuroxime, ciprofloxacin, piperacillin, cefoperazone, ceftazidime, ofloxacin, sulbactam, nitrofurantoin, doxycycline, norfloxacin, and meropenem, and the MIC values are represented in Supplementary Table 1.

### Agar cup method

The ethanolic extracts of the rhizome of both the in vivo and ex vitro plants and the silver bio-nanoparticles derived from the plant extracts were investigated against seven pathogenic strains of MDR-UTI bacteria. The antimicrobial potency of ex vitro rhizome was significantly higher than that of in vivo rhizome. While the ex vitro rhizome exhibited maximum inhibition against *Pseudomonas aeruginosa* (isolate no. 42332) with *ZI* = 14.03 ± 0.04 mm, only 11.56 ± 0.04 mm was observed in the case of in vivo rhizome extract. The antibacterial potency of the silver bio-nanoparticles was even greater than those against all the (seven out of seven) pathogenic strains, with a maximum *ZI* = 16.93 ± 0.04 mm against *Pseudomonas aeruginosa* (isolate no. 42332) (Table 2). Hence, it can be stated that the silver bio-nanoparticles are more competent as antimicrobics against human pathogens, where conventional antibiotics fail to work.

**Table 2:** Comparative antimicrobial activity of in vivo and ex vitro *Kaempferia angustifolia* (ethanol extract) and silver nanoparticle against UTI clinical pathogen

Antibacterial materials	Concentration	Zone of inhibition (mm)						
		Pathogenic bacteria						
		Isolate no. 42571	Isolate no. 42423	Isolate no. 42269	Isolate no.43164	Isolate no. 42230	Isolate no.42332	Isolate no. 43546
Plant extract (in vivo) Ethanol extract (mg/ml)	0.75	8.56 ± 0.09	7.53 ± 0.04	7.56 ± 0.09	8.40 ± 0.00	6.16 ± 0.12	9.33 ± 0.12	7.10 ± 0.08
	3.0	10.23 ± 0.20	10.1 ± 0.16	8.96 ± 0.04	10.06 ± 0.09	9.23 ± 0.09	10.96 ± 0.04	8.96 ± 0.04
	6.0	10.56 ± 0.09	11.53 ± 0.04	10.56 ± 0.04	11.10 ± 0.08	10.56 ± 0.09	11.56 ± 0.04	9.66 ± 0.04
Plant extract (ex vitro) Ethanol extract (mg/ml)	0.75	11.33 ± 0.04	10.53 ± 0.04	9.90 ± 0.08	10.23 ± 0.09	9.43 ± 0.09	10.00 ± 0.08	7.50 ± 0.00
	3.0	13.90 ± 0.08	13.86 ± 0.09	11.03 ± 0.12	13.46 ± 0.04	11.10 ± 0.14	12.93 ± 0.04	9.06 ± 0.04
	6.0	14.26 ± 0.12	15.13 ± 0.16	12.33 ± 0.20	14.03 ± 0.12	14.00 ± 0.08	14.03 ± 0.04	9.90 ± 0.08
Nanoparticle (μg/ml)	10	10.06 ± 0.09	12.4 ± 0.04	9.73 ± 0.04	11.40 ± 0.00	11.96 ± 0.12	11.30 ± 0.08	9.16 ± 0.09
	25	12.86 ± 0.04	15.03 ± 0.01	11.63 ± 0.12	13.93 ± 0.04	15.90 ± 0.08	15.76 ± 0.12	11.83 ± 0.12
	50	14.73 ± 0.16	16.60 ± 0.00	12.93 ± 0.04	15.10 ± 0.08	16.26 ± 0.12	16.93 ± 0.04	12.40 ± 0.12
Silver nitrate (μg/ml)	10	-	6.76 ± 0.06	-	-	7.4 ± 0.21	8.06 ± 0.09	-
	20	5.46 ± 0.12	7.96 ± 0.03	5.83 ± 0.04	8.23 ± 0.24	8.3 ± 0.14	9.53 ± 0.30	-
	50	6.96 ± 0.12	9.1 ± 0.04	7.13 ± 0.12	10.16 ± 0.23	9.13 ± 0.12	10.23 ± 0.16	5.46 ± 0.16

Isolate no. 42571, *Escherichia coli*; isolate no. 42423, *Escherichia coli*; isolate no. 42269, *Klebsiella pneumoniae*; isolate no. 43164, *Klebsiella pneumoniae*; isolate no. 42230, *Pseudomonas aeruginosa*; isolate no. 42332, *Pseudomonas aeruginosa*; isolate no. 43546, *Staphylococcus saprophyticus*

### MIC and MBC

MIC and MBC also confirmed that the antimicrobial potency of silver bio-nanoparticle is much better than in vivo and ex vitro extracts of the plant. The bio-nanoparticle has better inhibitory potency than a plant (MIC and MBC of 2.50 and 5.00 μg/ml) against isolate no. 42423. However, the result obtained from the rhizome is not negligible but more efficacious than the commonly used antibiotics. If we compare the results of in vivo and ex vitro plants, the ex vitro plant shows more efficiency by providing MIC and MBC of 0.12 and 0.25 mg/ml as compared to the in vivo plants with 0.75 and 1.00 mg/ml, respectively, against the same pathogen *Escherichia coli* (isolate no. 42423) (Table. 3).

**Table 3:** MIC and MBC of ethanol extracts of *Kaempferia angustifolia* (in vitro and ex vitro) along with nanoparticles

Microorganism		In vivo plant ethanol extract (mg/ml)		Ex vitro plant ethanol extract (mg/ml)		Nanoparticle (μg/ml)	
Type	ID	MIC	MBC	MIC	MBC	MIC	MBC
Pathogenic bacteria (multidrug-resistant strains of urinary tract infection)	Isolate no. 42571	1.00	1.75	0.50	0.75	5.25	6.50
	Isolate no. 42423	0.75	1.00	0.12	0.25	2.50	5.00
	Isolate no. 42269	1.25	1.75	0.75	0.75	5.75	7.25
	Isolate no. 43164	1.00	1.25	0.50	0.75	5.50	6.50
	Isolate no. 42230	1.25	2.00	0.50	0.75	7.25	10.00
	Isolate no. 43546	2.00	2.75	1.25	1.75	5.50	5.50
	Isolate no. 42332	0.75	1.50	0.75	1.00	7.75	9.50

Isolate no. 42571, *Escherichia coli*; isolate no. 42423, *Escherichia coli*; isolate no. 42269, *Klebsiella pneumoniae*; isolate no. 43164, *Klebsiella pneumoniae*; isolate no. 42230, *Pseudomonas aeruginosa*; isolate no. 42332, *Pseudomonas aeruginosa*; isolate no. 43546, *Staphylococcus saprophyticus*

## Discussion

Antibiotic resistance is a challenging phenomenon that makes bacteria multidrug resistant and tougher to extirpate and combat. The production of excess amounts of extracellular, polymeric substances makes them more resistant to antibiotics [29]. Plant-mediated synthesis of bio-nanoparticle (i.e. phytogenic nanoparticles) is environmentally benign and effective against different groups of pathogens [30–33]. Phytogenic nanoparticles are more potent and less toxic compared to chemically synthesized nanoparticles. The non-toxic nature of these bio-nanoparticles makes them advantageous and suitable for the use in medical studies [34–36]. The metallic silver itself is known to be a potential antimicrobial agent from the very past. The Ag^+^ ions in silver nanoparticles make them more effective against microbes. The silver nanoparticle can prevent bacterial infection by inhibiting cell wall synthesis and creating a hole between them [37].

In the present study, silver bio-nanoparticle, synthesized from the extracts of *K*. *angustifolia*, showed a wide range of antimicrobial activity against all seven multidrug-resistant human uropathogens. A similar activity of silver nanoparticles against uropathogens was reported by Rahuman et al. (2021) [38], but their ‘test pathogens’ were not multidrug resistant. The antimicrobial activity of silver nanoparticles against 11 gram-positive uropathogens was previously examined by Mishra and Padhy (2018) [39]. The effectivity of silver nanoparticles against eight multidrug-resistant human respiratory tracts infecting pathogen was previously reported in our previous study [40]. The present outcome revealed six out of eight gram-negative bacterial strains are more sensitive than two gram-positive strains to the silver bio-nanoparticles. The Ag^+^ ions in nanoparticles are known to have versatile activities, including inhibition of electron transport chain, inhibition of DNA replication, and membrane interaction, and causing damage to both DNA and RNA [11]. It can be hypothesized that the bio-nanoparticles are more effective against gram-negative than gram-positive bacteria, because of their differing cell wall structure. The lipopolysaccharide molecules, present in the cell walls of the gram-negative bacteria, carry a negative charge and hence have a higher affinity for the positive Ag^+^ ions of the nanoparticles, leading to a buildup and increased uptake of ions, which then cause intracellular damage.

The present manuscript is based on the biocatalysis of silver salt to silver nanoparticles by using plant rhizome extract. The size and shape of the bio-nanoparticles are impactful parameters for their antimicrobial activity [41]. In the present study, we found that the sizes of the silver bio-nanoparticles ranged from 10 to 15 nm, which are very useful for killing microbes. Some of the previous studies suggested that nanoparticles above 50 nm are less effective than smaller particles [42]. The shape of the nanoparticles also plays a vital role in killing bacteria. According to Pal et al. (2007), the truncated triangular-shaped nanoparticles exhibit maximum efficiency in controlling pathogenic bacteria [43]. The presently described method also exhibited different shapes, including truncated triangular and several others like spherical, rod, and oval-shaped silver bio-nanoparticles. However, the present study revealed that the assembly of different shapes of bio-nanoparticles contributed greatly toward their toxicity against uropathogens. The present findings corroborate with a previous study where versatility in the nanoparticle’s shape causes the maximum inhibition against the pathogens [44].

During the synthesis of silver bio-nanoparticle from *K. angustifolia*, the aggregative nature of the bio-nanoparticle was observed. The colloidal nature of the nanoparticles has a catalytic activity that can destabilize the enzymes of the microorganisms [45]. Few previous attempts have been done in synthesizing silver nanoparticles by using other species of Zingiberaceae plants like *Zingiber officinale*, *Curcuma amada*, *Curcuma longa, Kaempferia galanga*, *Kaempferia rotunda*, and *Kaempferia parviflora* [15, 16, 46–48]. However, according to our knowledge, this is the first attempt for the synthesis of silver nanoparticles and optimization against pathogenic strains from underutilized medicinal species of *K. angustifolia*. In addition to the bio-nanoparticle synthesizing potentiality, the plant extract of *K. angustifolia* also exhibited significant pathogen-killing properties. Due to resistance to common antibiotics, physicians lose their patience and prescribe a next-generation antibiotic ardently. As a result, pathogenic as well as inhabitat microflora also develop resistance to next-generation antibiotics.

The presence of divergent phytocompounds in crude plant extracts displays a synergistic effect against microbes; as a result, plants exhibit stronger antimicrobial activity compared to a single drug [49]. Previous reports on the antimicrobial activity of *K. angustifolia* are mainly based on the essential oil of this plant [26]. The percentage yield of essential oil is 0.26% of fresh weight, which is very low, and a large amount of rhizome is required for hydro distillation. On the other hand, the crude rhizome extract is obtained in a higher amount of about 12.6% of fresh weight, compared to the essential oil. Hence, the rhizome extract can be a better alternative as a source of antimicrobics against pathogens. According to Tang et al. (2014), *K. angustifolia* extract did not display any antimicrobial activity against the microbes they used in their study. On the contrary, the present study confirmed that both the ex vitro and in vivo extracts of rhizome of this plant have moderate to high inhibition potentiality against all the pathogens used [7], ex vitro being the more effective one can be considered as a prospective agent for killing MDR UTI pathogens.

## Conclusion

The present manuscript describes the rapid synthesis of silver bio-nanoparticle through the catalysis of silver nitrate salt by rhizome extract of the plant *K. angustifolia*. This is probably the first report of silver nanoparticle production using this underutilized medicinal plant. The presently described method will be helpful not only for the large-scale bioproduction of the silver nanoparticle but also for the production of different other metal nanoparticles. The crude rhizome extract and phytogenic silver bio-nanoparticle exhibited substantial antimicrobial activities against multidrug-resistant human urinary tract infecting pathogenic strains. However, further study on other groups of pathogens and identification of phytocompounds from the rhizome extracts and also from an essential oil are going on employing the in vitro*,* in vivo, and ex vitro plants of *K. angustifolia* and will be communicated later.

## Supplementary Information

**Additional file 1: Supplementary Table 1**. Antibiotic sensitivity pattern obtained from Vitek2 Compact system.

## Data Availability

The authors declare that all data supporting the findings of this study are included within the article.

## Abbreviations

MDR: Multidrug-resistant
UTI: Urinary tract infection
DLS: Dynamic light scattering
EDS: Electron dispersion spectroscopy
HRTEM: High-resolution transmission electron microscope
DMSO: Dimethyl sulfoxide
MHA: Mueller-Hinton agar
MIC: Minimum inhibitory concentration
MBC: Minimum bactericidal concentration
