# Supplementary material for: Phytogenic silver nanoparticles from tissue-cultured Kaempferia angustifolia — an underutilized medicinal herb: a comparative antibacterial study on urinary pathogens
Source: J Genet Eng Biotechnol. 2022 Sep 8;20:131. doi: 10.1186/s43141-022-00414-4 (PMC9458813; doi:10.1186/s43141-022-00414-4)
Supplement: Supplementary file 1 — Additional file 1: Supplementary Table 1. Antibiotic sensitivity pattern obtained from Vitek2 Compact system. [file 43141_2022_414_MOESM1_ESM.docx]

| Supplementary table 1. Antibiotic sensitivity pattern obtained from Vitek2 Compact system. | | | | | | | | | | | | | | | | | |
| --- | --- | --- | --- | --- | --- | --- | --- | --- | --- | --- | --- | --- | --- | --- | --- | --- | --- |
| Isolated bacteria  (Isolate No.) | Vitek2 Result | Antibiotics | | | | | | | | | | | | | | | |
|  |  | Amoxiline | Amikacin | Aztreonam | Ceftriaxone | Cefuroxime | Ciprofloxacin | Piperacillin | Cefoperazone | Ceftazidime | Ofloxacin | Sulbactam | Nitrofurantoin | Doxycycline | Norfloxacin | Meropenem |  |
| *Escherichia coli*  (42571) | MIC (µg/ml) | ≥32 | 4 | ≥16 | ≥64 | ≥ 32 | ≥4 | ≤4 | ≥64 | ≥16 | ≥8 | ≤4 | ≤16 | ≤2 | ≥16 | ≤1 |  |
|  | Interpretation | R | S | R | R | R | R | S | R | R | R | S | S | S | R | S |  |
| *Escherichia coli*  (42423) | MIC (µg/ml) | ≤8 | 8 | ≥16 | ≥64 | ≥ 32 | **−** | ≤4 | ≤4 | ≥16 | ≥8 | ≥16 | ≥128 | ≥16 | ≥16 | ≤0.5 |  |
|  | Interpretation | S | S | R | R | R | − | S | S | R | R | R | R | R | R | S |  |
| *Klebsiella pneumoniae* (42269) | MIC (µg/ml) | ≥32 | ≥64 | ≥16 | ≥64 | ≥ 32 | 0.5 | ≥32 | ≥64 | ≥16 | 4 | ≥16 | **−** | ≤1 | 8 | 2 |  |
|  | Interpretation | R | R | R | R | R | I | R | R | R | I | R | − | S | I | I |  |
| *Pseudomonas aeruginosa* (42230) | MIC (µg/ml) | − | ≤0.5 | ≥32 | − | 8 | 1 | ≤4 | − | ≥16 | ≥32 | ≥8 | ≥16 | ≤2 | ≤4 | ≤2 |  |
|  | Interpretation | − | S | R | − | I | I | S | − | R | R | R | R | S | S | S |  |
| *Staphylococcus saprophyticus* (43546) | MIC (µg/ml) | ≤4 | ≤16 | ≤8 | ≤2 | ≥16 | ≤1 | ≤2 | ≤4 | ≤4 | ≤1 | 4 | ≥16 | ≥32 | 8 | ≤0.25 |  |
|  | Interpretation | S | S | S | S | R | S | S | S | S | S | I | R | R | I | S |  |
| *Klebsiella pneumoniae* (43164) | MIC (µg/ml) | ≥32 | ≥64 | ≥16 | ≥64 | ≥ 32 | ≥4 | ≥32 | ≥64 | ≥16 | ≥8 | − | ≥128 | ≥16 | − | ≥4 |  |
|  | Interpretation | R | R | R | R | R | R | R | R | R | R | − | R | R | − | R |  |
| *Pseudomonas aeruginosa* (42332) | MIC (µg/ml) | 4 | ≤0.5 | ≥32 | ≥64 | ≥32 | ≥2 | ≥32 | ≥16 | ≤2 | ≤1 | ≤4 | 4 | ≤2 | ≥16 | 4 |  |
|  | Interpretation | I | S | R | R | R | R | R | R | S | S | S | I | S | R | I |  |
| MIC = Minimum Inhibitory Concentration, R= Resistant, S= Sensitive, I= Intermediate, ‘−’= Not tested  Ox = Oxidase test, Cat = Catalase test, Ind = Indole test, MR = Methyl Red test, Cit = Citrate test, VP = Voges-Proskauer test, H_2_S = Hydrogen sulphide | | | | | | | | | | | | | | | | | |

**Supplementary material for**

**Antibiotic sensitivity pattern of seven UTI pathogenic bacterial strains against different 15 antibiotics**
